# Cost analysis of three-dimensional radiation therapy versus intensity-modulated chemoradiotherapy for locally advanced cervical cancer in Peruvian citizens

**DOI:** 10.3332/ecancer.2023.1531

**Published:** 2023-04-20

**Authors:** José Fernando Robles Díaz

**Affiliations:** Regional Institute for Neoplastic Diseases, Central Region, Concepción, Junín 12126, Peru and Los Andes Peruvian University, Huancayo 12002, Peru

**Keywords:** costs and cost analysis, cervical neoplasia, chemoradiotherapy, intensity-modulated radiation therapy, simultaneous integrated boost

## Abstract

**Background and objectives:**

The standard treatment for locally advanced cervical cancer (CC) is chemoradiotherapy (CTRT) followed by high-dose-rate brachytherapy (HDRBT). The ideal scenario would be under novel intensity-modulated radiation therapy (IMRT) volumetric-modulated arc therapy (VMAT) radiation techniques over three-dimensional (3D) radiation therapy. However, radiotherapy (RT) centres in low- and middle-income countries have limited equipment for teletherapy services like HDRBT. This is why the 3D modality is still in use. The objective of this study was to analyse costs in a comparison of 3D versus IMRT versus VMAT based on clinical staging.

**Materials and methods:**

From 02/01/2022 to 05/01/2023 a prospective registry of the costs for oncological management was carried out for patients with locally advanced CC who received CTRT ± HDRBT. This included the administration of radiation with chemotherapy. The cost associated with patient and family transfers and hours in the hospital was also identified. These expenses were used to project the direct and indirect costs of 3D versus IMRT versus VMAT.

**Results:**

The treatment regimens for stage IIIC2, including 3D and novel techniques, are those with the highest costs. The administration of 3D RT for IIIC2 and novel IMRT or VMAT techniques, is $3,881.69, $3,374.76, and $2,862.80, respectively. The indirect cost from stage IIB to IIIC1 in descending order is IMRT, 3D and VMAT, but in IIIC2 the novel technique regimens reduce by up to 33.99% compared to 3D.

**Conclusion:**

In RT centres with an available supply of RT equipment, VMAT should be preferred over IMRT/3D since it reduces costs and toxicity. However, in RT centres where demand exceeds supply in the VMAT technique planning systems, the use of 3D teletherapy over IMRT/VMAT could continue to be used in patients with stage IIB to IIIC1.

## Introduction

According to GLOBOCAN 2020, cervical cancer (CC) ranks fourth with an incidence of 13.3 and second with an incidence of 22.2 in women throughout the world and Peru, respectively. It has an incidence-mortality rate of 14.9–7.6 and 22.5–11.5 per 100,000 people on a Latin American and national level, respectively [1], thus making it one of the most costly oncologic pathologies for the health system. Compared with countries with a high level of resources, like the United States or Western European countries, developing countries like Peru and Indonesia, which have limited monetary and educational resources in healthcare, have the highest CC incidence and mortality rates [2]. CC is the fourth most common type of cancer in women under 45 [3], yet the average at diagnosis with CC is 51 [2].

Treatment for invasive CC is stratified by the stage of the disease, which is determined by the International Federation of Gynaecology and Obstetrics (FIGO) as well as the Tumor, Nodal, Metastasis staging system of the American Joint Committee on Cancer/Union of International Cancer Control [4, 5]. These criteria were last updated in 2018 and 2021, respectively [6]. Chemoradiotherapy (CTRT), which involves platinum-based chemotherapy (CT) concurrent with external beam radiotherapy (RT) followed by high dose-rate brachytherapy (HDRBT), is the standard treatment as confirmed by Phase III trials and meta-analysis [7–9]. The use of intensity-modulated RT and three-dimensional (3D) image-guided adaptive HDRBT has improved the results and reduced lesions in the surrounding organs [10]. 

Up until now, there have been few economic evaluation reports available on 3D radiation versus novel techniques like intensity-modulated radiation therapy (IMRT) or volumetric-modulated arc therapy (VMAT) in countries with a low-to-high human development index (HDI). In a country where the health system has limited funding, it is thereby important to discuss on radiation in CC using cost analysis [11]. As such, the objective of this study was to conduct a cost analysis by comparing the 3D technique versus novel techniques in patients with advanced CC.

## Materials and methods

From 02/01/2022 to 05/01/2023 the clinical features of patients with CC who received radical treatment with CTRT were prospectively recorded using medical histories. Transportation expenses were collected using patient questionnaires on their daily expenses for travelling to the institute. Medical costs were obtained from their medical records and pharmacy archives as well as those for facility-equipment stocks. The medications and supplies used in the preparation and administration of CT and RT were identified during this process. Also, the health professionals involved were interviewed to find out about the procedure times. This study was approved in advance by the Institutional Ethics Committee. Only patients who provided comprehensive data and signed the informed consent were analysed.

### Estimated cost

A spreadsheet was made to project the cost of CC treatment based on FIGO clinical stages. The direct and indirect costs were included in Peruvian currency (sol, S/.) as well as in their American dollar equivalent ($). The direct medical costs were recorded with the monetary equivalent of the healthcare professionals’ working hours, equipment, facilities, medications and supplies used. The indirect costs associated with the time patients and relatives spent in the facility, including travel time from their homes were also included.

This data analysis was conducted on patients being treated who had stage IIB–IVB, had completed CTRT treatment and were thereby granted full costs access. The type of radiation technique was divided into two groups; those who began with 3D and those who began with the novel techniques (IMRT/VMAT).

### Treatment regimen based on staging

All patients received CTRT as the initial treatment. The CT sensitiser was a weekly 40 mg/m^2^ dose of Cisplatin on an outpatient basis. There was a difference in the initial teletherapy modalities. This was either through 3D or novel techniques with the Synergy Platform^®^ and Full de Elekta^®^ linear accelerators (LINAC), respectively. HDRBT was administered at all stages except IVA using high dose-rate MicroSelectron equipment with iridium source by Elekta^®^. 3D HDRBT consisted of a dose of 7 Gy in 4 sessions over 2 weeks.

- Stage IIB/IIIA/IIIB: Pelvis 3D CTRT at a dose of 5,000 cGy in 25 sessions over 5 weeks followed by HDRBT as the first option (3D CTRT25 + HDRBT). Novel technique pelvis QTRT25 CTRT at a dose of 5,000 cGy in 25 sessions over 5 weeks followed by HDRBT as the second option (IMRT CTRT25 + HDRBT/VMAT CTRT25 + HDRBT).
- Stage IIIC1: 3D pelvis CTRT at a dose of 5,600 cGy in 28 sessions over 6 weeks followed by HDRBT as the first option (3D CTRT28 + HDRBT). Novel technique pelvis CTRT at a dose of 5,880 cGy in 28 sessions over 6 weeks followed by HDRBT as the second option (IMRT CTRT28 + HDRBT/VMAT CTRT28 + HDRBT).
- Stage IIIC2: 3D pelvis CTRT at a dose of 5,600 cGy in 28 sessions over 6 weeks followed by HDRBT, before finishing with novel technique para-aortic (PA) at a dose of 5,500 cGy in 25 sessions over 5 weeks as the first option (3D CTRT28 + HDRBT + VMAT + CT25). Novel technique pelvic and para-aortic (PPA) CTRT at a dose of 5,880 cGy in 28 sessions over 6 weeks, followed by HDRBT as the second option (IMRT CTRT28 + HDRBT/VMAT CTRT28 + HDRBT).
- Stage IVA: 3D pelvis CTRT at a dose of 5,600 cGy in 28 sessions over 6 weeks, followed by a dose of 2,000 cGy 3D in 5 sessions over 1 week was the only option for the residual tumour. (3D CTRT28 + 3D RT5). No comparison was made with novel techniques and there are no HDRBT applicators for bladder and/or rectum involvement.
- Stage IVB: 3D pelvis and groin CTRT with a dose of 6,000 cGy in 30 sessions over 6 weeks and HDRBT (3D CTRT30 + HDRBT) was the only option. No comparison was made with the novel techniques, only patients who had inguinal node metastasis were entered. Those that had vesical or rectal involvement as well as PA or visceral metastases were excluded.

### Direct cost

The calculation was based on the cost of medications and supplies for CTRT [12], the monetary equivalent for the equipment and facilities [13] usage time in consultation, simulation, treatment volume delimitation, planning, HDRBT preparation as well as CT administration procedures. It analysed healthcare professional costs proportional to the monthly salary for the time involved in procedures [14]. Toxic effects were not analysed as most patients do not have any significant adverse effects.

### Indirect cost

This calculation was based on patients and companions. It included the monetary equivalent for the time of their admission into and discharge from the procedure facility. The average cost of transport from their home to the facility was added. Our analysis included costs associated with companions because the therapy that studies participants received often affects the patient’s independence. The productivity per hour worked reference value for the central macro-region [15] was used to calculate the loss of productivity due to being out of work for the patient and relatives alike.

### Statistical analysis

All data and statistical analyses were conducted using SPSS version 25. Descriptive statistics are presented as means or proportions.

## Results

The research ended in on 05/01/2023 by filing 44 patients on CTRT, who had comprehensive data for the study. Details for patients and their companions are presented in Table 1. Moreover, the unit cost of the resources used for the comparative projections is presented in Table 2.

The regimens 3D CTRT25 + HDRBT, 3D CTRT28 + HDRBT, 3D CTRT28 + HDRBT + VMAT RT25, 3D CTRT28 + 3D RT5, 3D CTRT30 + HDRBT, IMRT CTRT25 + HDRBT, VMAT CTRT25 + HDRBT, IMRT CTRT28 + HDRBT, VMAT CTRT28 + HDRBT, IMRT CTRT28 + HDRBT (IIIC2) and VMAT CTRT28 + HDRBT (IIIC2), have a direct cost of $3 048.85, $3 399.33, $4 856.47, $2 790.87, $3 501.28, $3 574.12, $3 208.43, $4 001.62, $3 592.05, $4 349.55 and $3 837.58, respectively. The treatment regimens for stage IIIC2, including 3D and novel techniques, are those with the highest costs. The administration of 3D RT for IIIC2 and novel IMRT or VMAT techniques, is $3,881.69, $3,374.76 and $2,862.80, respectively. Planning increased in value from $25.3 to $58.83 when novel technique therapy began over 3D. Also, all first option sessions (28 3D pelvis sessions and 25 PA VMAT sessions) are almost double the cost of 28 PPA VMAT sessions yet much lower than PPA IMRT (Table 3).

The indirect cost from stage IIB to IIIC1 in descending order is IMRT, 3D and VMAT, but in IIIC2 the novel technique regimens reduce by up to 33.99% compared to 3D. The resulting costs of lost household productivity for 3D CTRT25 + HDRBT, 3D CTRT28 + HDRBT, 3D CTRT28 + HDRBT + VMAT RT25, 3D CTRT28 + 3D RT5, 3D CTRT30 + HDRBT, IMRT CTRT25 + HDRBT, VMAT CTRT25 + HDRBT, IMRT CTRT28 + HDRBT, VMAT CTRT28 + HDRBT, IMRT CTRT28 + HDRBT (IIIC2) and VMAT CTRT28 + HDRBT (IIIC2) are $218.47, $241.43, $346.63, $252.46, $249.99, $219.77, $217.17, $242.88, $239.98, $245.06 and $241.43, respectively (Table 3).

**Figure 1.**
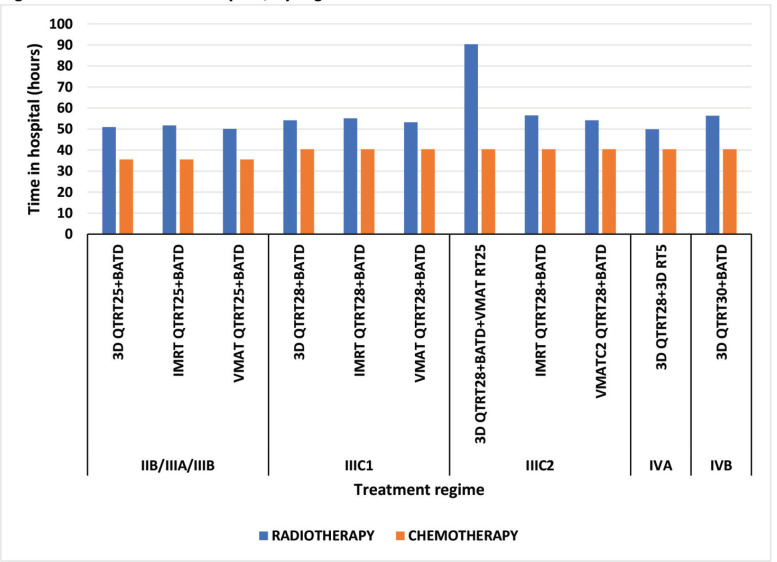
Patient time in hospital, by regimen.

The schemes that comprise a higher number of sessions, due to the additional administration involved with using CT and the IMRT technique, result in the patient staying in the hospital for more than 96 hours; which is why the 3D CTRT28 + HDRBT + VMAT RT25 (IIIC2) and IMRT CTRT28 + HDRBT (IIIC2) schemes have the longest hospital stays – 131 and 97 hours, respectively (Figure 1).

**Figure 2.**
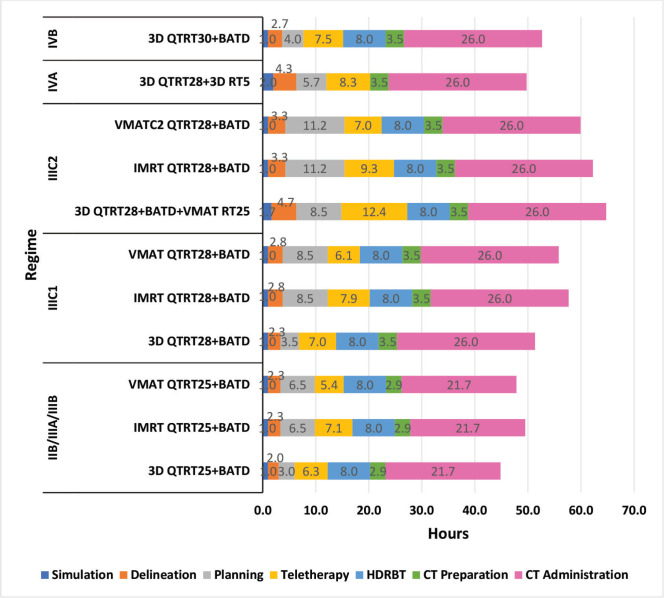
Comparison of procedure time, by regime.

Regarding procedure delivery, from stage IIB to IIIC1, the 3D and VMAT techniques are usually similar with values between 44 and 56 hours; however, the IMRT technique is usually longer than both techniques by at least 5 hours. The special techniques planning is usually longer, but this is offset by the reduced teletherapy time for VMAT, which is cut from 6.3 to 5.4 hours, 7.0 to 6.1 hours and 12.4 to 7 hours for stages IIB/IIIA/IIIB, IIIC1 and IIIC2, respectively (Figure 2).

## Discussion

The institute is financed by the state, located 284 km from Lima and responsible for cancer treatment in the central part of the country. Patients with CC have comprehensive health insurance, which means that the state subsidises the direct costs of medical care. For CTRT and HDRBT, the hospital covers the cost of medication; associated supplies, equipment and infrastructure; and the work of healthcare professionals. However, finance and equipment are less than the demand, so it is pertinent to make managerial decisions, supported by efficiency and in addition to the associated costs, as this will allow more cancer treatments to be offered [16, 17].

It is expected that, by 2040, the increased incidence and mortality will be disproportionately higher in low (LIC) and middle-income countries (MIC), with an estimated 72% higher incidence and 76% higher mortality in countries with low-to-medium HDI (Peru or Indonesia). This imbalance in the cancer burden is compounded by current disparities in resources for combatting cancer, which must be addressed as a priority [18, 19].

CC is the fourth most common type of cancer and the fourth leading cause of cancer mortality in women worldwide [20]. Radical RT plays an important role in the definitive treatment of locally-advanced disease, with 8-year survival rates of up to 67% when combined with concurrent CT [21]. Even in retrospective analysis, the addition of local RT at stage IVB seems to confer better results in specific survival [22, 23], which is why at our institution the treatment is offered to those with inguinal metastasis. The therapy administered with an HDRBT boost is the standard of care [24]; IMRT or VMAT have become standard therapy methods, compared to 3D [25]. The dose is determined and administered via new technologies, which use inverse optimisation algorithms, as they are consistent with the planned target area and provide a more even distribution [26].

There are many published studies on CTRT regimens that have been widely used in clinical practice. However, there is limited research on economic analysis. This is the first Latin American study that analyses the costs separated by clinical stage, comparing irradiation techniques. Unlike other studies that are based on annual projections by incidence [27], and others that only focus on the cost of human resources and infrastructure [28], due to the nature of the study it has aimed to determine, in detail, both direct and indirect costs, not overlooking micro costs [29, 30].

The direct cost increases in line with the clinical stage from IIB to IIIC2 (Table 3). However, at stage IVA there is a decrease because HDRBT is not used, due to an absence of suitable applicators at the institution. This trend is comparable to the study by Nguyen *et al* [31], which shows that in hospitals in central Vietnam, the medical costs for the treatment of CC increase as the cancer progresses to the later stages. In addition, Granados-Garcia *et al* [32] report that the medical cost for stages I–IV was between $4,738 and $6,058. In the study, RT represented 77% of the direct cost; this data was consistent with the study by Santos *et al* [33] of Brazilian women with CC regardless of the stage, RT and CT represented a cost of $1,491 and $1,069, respectively.

Furthermore, our study shows that the direct cost is markedly reduced by starting irradiation with VMAT in clinical stage IIIC2, unlike the rest of the clinical stages, as starting the treatment with 3D only sequentially irradiates the pelvis and PA region, resulting in an increased cost, to meet the volumes of PPA nodes recommended by EMBRACE II [34]. Nevertheless, the inclusion of PA volumes has been shown to increase the risk of acute and delayed intestinal toxicity [35, 36]. This is why, at the institution at stage IIIC2, the pelvic segment is treated first and then the PA segment, increasing the number of sessions and, as a result the number of procedures [37]. Urban *et al* [38], based on patients at clinical stage IA1 to IIIC2, revealed that patients undergoing definitive teletherapy with an HDRBT boost reported less late and subacute gastrointestinal toxicity when treated with IMRT/VMAT, compared to 3D. So there is a clinical benefit, in addition to an economic benefit as demonstrated by the study, as there is a marked decrease in the cost of teletherapy when using VMAT rather than IMRT or 3D.

Regarding the intra-hospital procedure time, more time is generally required during the administration of CT, which is only considered concurrent, but not in the later stage of CTRT, given that for stage IIB to IVA, CTRT is more cost-effective than CTRT followed by adjuvant CT [39]. On the other hand, when the special technique is used, it requires a great deal more planning for the same complexity but shortens the teletherapy time for VMAT (Figure 2). This is due to advanced technology also carrying a corresponding computational burden, which considerably increases the total planning time. According to statistics, radiotherapists spend an average of 4 hours delineating the target volume plan and organs at risk, and it can also be delayed further by some complex illnesses. After this, the medical physicists formulate an RT plan which complies with treatment standards, which takes approximately 10 hours per patient [40, 41]. A large amount of time required for planning inevitably leads to a delay in treatment, which then affects the quality of treatment and the prognosis of the patients [42]. In the future, based on steep learning networks and optimisation algorithms, like the voxel dose restriction optimisation model or setting up predicted Dose Volume Histograms-aided targets, it will allow the development of an automated planning system that will ultimately serve the doctors and physicists, balancing the cost of time and precision [41].

Our study shows that indirect costs are lower for all stages using VMAT. The difference in the loss of productivity and the use of transportation is notable (Table 3). This is due to the reduction of hospital visits, and the shorter time involved in each teletherapy session; consequently, the accumulated time is less than with IMRT or 3D (Figure 1). These are the only categories evaluated for indirect cost, which represents at most 26.17% and 17.27% of the annual household income ($3,586.32) [15] with the 3D QTRT28 + BATD + VMAT RT25 and VMAT QTRT28 + BATD schemes, respectively (Table 3). This result is worrisome, due to the risk of incurring catastrophic costs and increasing barriers to compliance with treatment [43–45].

At present, the option exists to reduce the number of BATD sessions [46] but at the teletherapy level, it is not recommended to use hypofractionation in order to reduce the number of sessions as in other pathologies [8, 17]. However, the future option exists to offer hypofractionation of 15 sessions followed by 4 BATD applications in women with CT contraindications such as advanced illness with secondary renal dysfunction and hydronephrosis related to the tumour, borderline cardiac function; and fragile patients [47]. Similarly, by means of phase II trials NCT04070976 and NCT04583254, the efficacy and safety of hypofractionated doses are studied in CTRT. Currently, the recommended splits are between 25 and 28 sessions of special techniques with simultaneous integrated boost, which is impossible to accomplish this in a country with a marked deficit of RT equipment [48]. The international atomic energy agency ideally recommends four units of RT per million people, with a minimum of at least 1.5 units per million. However, currently, one unit of RT helps 0.12 million people in HIC, in contrast to 1 million in MIC and 5 million in LIC [19, 49]. The institute has an assigned population of approximately 3,681,400 with only two linear particle accelerators; therefore, it cannot provide special technical treatment to all of the patients due to the high demand. Replicate this scenario on a national level with a population of 33.72 million and only 59 sets of teletherapy equipment and 18 sets of brachytherapy (BT) equipment all of which are gathered in the capital city. This also occurs in China, India and Argentina where a disparity exists in the density of facilities within a country and only being able to receive treatment within a reasonable timeframe in modern cities [50–53]. 

In other studies of non-gynaecological pathology costs, they do simultaneous variation in the treatment time, capacity utilisation rates and the number of LINAC staff, obtaining a cost reduction [54]. The analysis was carried out with the minimum number of staff necessary for LINAC, two medical technologists. Staffing costs could thus not have been reduced further. Perhaps using another LINAC that optimises treatment speed through gantry mobility and MLC motion, such as the Varian^®^ Halcyon [55, 56], could significantly lower teletherapy costs. Similarly, the limitation of the special techniques planning is that it only has two RT planning systems, running 12 hours a day, for the whole service. Consequently, the only way to increase the supply of special technical plans and LINAC services would be to recruit more professionals in order to increase the working hours and teletherapy supply capacity from 32 to 48 patients per day, but this involves more hours of doing radiation, which has a direct outcome of reducing the operational lifespan of LINAC [57, 58]. 

The limitations of the study are: First, we made some simplifying assumptions regarding the natural history and treatment of the disease. Second, the limited number of patients could have different costs due to social status. Third, it is possible that we did not account for differences in delayed toxic effects as follow-up data is limited. However, the total rate of these effects seems lower through special techniques according to clinical trials.

## Conclusion

Our study is one of the few to evaluate the costs of 3D and special technique treatments that have incorporated patient costs, direct costs, added over time and the indirect cost to patients and their households. Moreover, this is the first report that includes clinical and economic oncologic data from a Peruvian public hospital.

In RT centres with a positive supply over demand of modern LINAC equipment, VMAT should be preferred over IMRT/3D as it lowers costs and toxicity, but in RT centres where demand exceeds the supply of planning systems with VMAT techniques, patients with stages IIB to IIIC1 cancer could continue making use of 3D teletherapy over IMRT/VMAT for the short period of time during the planning process, but VMAT is preferable for stage IIIC2 patients, as it saves hospital resources and indirect costs.

## Conflicts of interest declaration

There are no conflicts of interest in this research.

## Project funding

No funding has been received.

## Figures and Tables

**Table 1.** Characteristics of patients and companions.

Category	*N*	%	Mean	Median	SD
Patient					
Age	44		51.41	52.00	4.41
Body area	44		1.49	1.50	0.07
Companion					
Yes	44	100.00			
No	0	0.00			
Age	44.00		27.00	20.00	10.80
Working	28	73.68			
Unemployed	16	42.11			
Monthly income household^$^	44		293.53	271.58	76.49

One American Dollar $ is equivalent to 3.80 Peruvian Sol (S/)

**Table 2.** RT and CT-related unit costs.

Phase	Resources	American dollars^$^
RT	Radiation oncologist per minute	0.23
	Medical technician per minute	0.14
	Nurse per minute	0.15
	Nursing technician per minute	0.08
	Medical physicist per minute	0.15
	MSCT simulator*	0.08
	Laser alignment system*	0.02
	Simulation infrastructure*	0.01
	Simulation equipment*	0.01
	Immobilisation device*	0.01
	Software*	0.06
	3D planning infrastructure and equipment*	0.01
	IMRT/VMAT planning infrastructure and equipment*	0.01
	Synergy platform linear accelerator*	1.39
	Synergy full linear accelerator*	2.16
	3D teletherapy infrastructure*	0.01
	IMRT/VMAT teletherapy equipment*	0.61
	IMRT/VMAT teletherapy infrastructure*	0.01
	BT infrastructure*	0.05
	BT equipment*	0.39
	Iridium source*	2.85
	Sodium diclofenac 75 mg Inj. per unit	0.04
	Capecitabine 0.5 mg tablet per unit	0.01
	Misoprostol 200 ucg per unit	0.58
	Foley catheter N° 14 French per unit￼	0.39
	Sterile glove per pair	0.58
	5 mL syringe per unit	0.04
	10 mL syringe per unit	0.04
	60 mL syringe per unit	5.00
	Sodium chloride 9% 1,000 mL per unit	0.65
	Iodovopidone 8.5% 1,000 mL per unit	3.95
	Iodopovidone 10% 1,000 mL per unit	3.95
	Lidocaine hydrochloride 2% gel per unit	0.59
	Sterile Gauze 10 × 10 cm per package	0.47
	Disposable speculum per unit	1.32
	Protective suit for 6 hours	1.81
CT	Medical oncologist per minute	0.23
	Pharmaceutical chemist per minute	0.16
	Nurse per minute	0.15
	Nursing technician per minute	0.08
	Cisplatin 50 mg Inj. per unit	4.66
	Ranitidine 50 mg Inj. per unit	0.09
	Dexamethasone 20 mg Inj. per unit	2.49
	Ondansetron 8 mg Inj. per unit	0.15
	Chlorphenamine 10 mg Inj. per unit	0.04
	Sodium chloride 9% 1,000 mL per unit	0.65
	Manitol 20% Inj. per unit	1.85
	Magnesium sulphate 200 mg Inj. per unit	0.17
	Omeprazole 20 mg cap per unit	0.02
	Metoclopramide 10 mg tab per unit	0.02
	Ondansetron 8 mg tab per unit	0.29
	Infusion set	1.80
	Heparin syringe	0.66
	20 mL syringe	0.10
	Sterile gloves	0.58
	Protective suit for 6 hours	1.81
	Saline solution 250 mL	0.89
	Sterile Gauze 10 × 10 cm per package	0.47
	Cotton swabs	0.08
	Surgical tape	0.06
	Alcohol	0.01

* Cost equivalent to one minute of utility

One American $ is equivalent to 3.80 Peruvian Sol (S/)

**Table 3.** Comparison of direct and indirect costs by treatment regimens.

DIRECT COST												
**SCHEME**		**Tridimensional**					**Special technique**					
**Treatment**	**Stage**	**IIB/IIIA/IIIB**	**IIIC1**	**IIIC2**	**IVA**	**IVB**	**IIB/IIIA/IIIB**		**IIIC1**		**IIIC2**	
	**Category**	**3D QTRT25 + BATD**	**3D QTRT28 + BATD**	**3D QTRT28 + BATD + VMAT RT25**	**3D QTRT28 + 3D RT5**	**3D QTRT30 + BATD**	**IMRT QTRT25 + BATD**	**VMAT QTRT25 + BATD**	**IMRT QTRT28 + BATD**	**VMAT QTRT28 + BATD**	**IMRT QTRT28 + BATD**	**VMATC2 QTRT28 + BATD**
RT	Consultation	22.27	22.27	44.54	27.83	22.27	22.27	22.27	22.27	22.27	22.27	22.27
	Simulation	78.57	78.57	144.08	157.14	78.57	78.57	78.57	78.57	78.57	78.57	78.57
	Delimitation	34.05	39.72	79.45	73.77	45.40	39.72	39.72	46.82	46.82	55.33	55.33
	Planning	36.26	42.30	107.97	68.49	48.35	76.96	76.96	101.14	101.14	133.37	133.37
	Teletherapy	1,127.91	1,263.26	2,527.23	1,488.84	1,353.49	1,606.81	1,241.12	1,799.62	1,390.05	2,106.80	1,594.84
	BT	978.42	978.42	978.42	0.00	978.42	978.42	978.42	978.42	978.42	978.42	978.42
	Subtotal	2,277.48	2,424.55	3,881.69	1,816.08	2,526.50	2,802.76	2,437.07	3,026.84	2,617.27	3,374.76	2,862.80
CT	Consultation	34.95	34.95	34.95	34.95	34.95	34.95	34.95	34.95	34.95	34.95	34.95
	CT preparation	283.89	396.80	396.80	396.80	396.80	283.89	283.89	396.80	396.80	396.80	396.80
	CT administration	452.53	543.04	543.04	543.04	543.04	452.53	452.53	543.04	543.04	543.04	543.04
	Subtotal	771.37	974.78	974.78	974.78	974.78	771.37	771.37	974.78	974.78	974.78	974.78
Total per patient (Peruvian Nuevo Sol (S/.)		11,585.62	12,917.47	18,454.60	10,605.29	13,304.88	13,581.67	12,192.05	15,206.17	13,649.80	16,528.27	14,582.81
Total per patient (Dollars)		3,048.85	3,399.33	4,856.47	2,790.87	3,501.28	3,574.12	3,208.43	4,001.62	3,592.05	4,349.55	3,837.58
**INDIRECT COST**												
**SCHEME**		**Tridimensional**					**Special technique**					
**Stage**		**IIB/IIIA/IIIB**	**IIIC1**	**IIIC2**	**IVA**	**IVB**	**IIB/IIIA/IIIB**		**IIIC1**		**IIIC2**	
**Category**		**3D QTRT25 + BATD**	**3D QTRT28 + BATD**	**3D QTRT28 + BATD + VMAT RT25**	**3D QTRT28 + 3D RT5**	**3D QTRT30 + BATD**	**IMRT QTRT25 + BATD**	**VMAT QTRT25 + BATD**	**IMRT QTRT28 + BATD**	**VMAT QTRT28 + BATD**	**IMRT QTRT28 + BATD**	**VMATC2 QTRT28 + BATD**
Loss of productivity due to consultation and treatment		134.62	147.20	203.53	140.59	150.58	135.92	133.32	148.66	145.75	150.84	147.20
Transportation		213.16	230.92	388.42	242.76	242.76	213.16	213.16	230.92	230.92	230.92	230.92
loss of household productivity		218.47	241.43	346.63	252.46	249.99	219.77	217.17	242.88	239.98	245.06	241.43
Total per patient (Peruvian Nuevo Sol (S/.)		2,151.74	2,354.30	3,566.60	2,416.06	2,444.65	2,161.60	2,141.88	2,365.35	2,343.26	2,381.91	2,354.30
Total per patient (Dollars)		566.25	619.55	938.58	635.81	643.33	568.84	563.65	622.46	616.65	626.82	619.55

3D QTRT25 + High Dose Rate Brachytherapy (BATD): Irradiation regimen of 25 sessions in 3D to the pelvis concurrent with CT, followed by 4 BATD applications. 3D QTRT28 + BATD: Irradiation regimen of 28 sessions in 3D to the pelvis concurrent with CT, followed by 4 BATD applications. 3D QTRT28 + BATD + VMAT RT25: Irradiation regimen of 28 sessions in 3D to the pelvis concurrent with CT, followed by 4 BATD applications and 25 VMAT sessions at RTP. 3D QTRT28 + 3D RT5: Irradiation regimen of 28 sessions in 3D to the pelvis concurrent with CT, followed by 5 sessions in 3D to residual tumour lesion. 3D QTRT30 + BATD: Irradiation regimen of 30 sessions in 3D to the pelvis concurrent with CT, followed by 4 BATD applications. IMRT QTRT25 + BATD: Irradiation regimen of 25 sessions in IMRT to the pelvis concurrent with CT, followed by 4 BATD applications. VMAT QTRT25 + BATD: Irradiation regimen of 25 sessions in VMAT to the pelvis concurrent with CT, followed by 4 BATD applications. IMRT QTRT28 + BATD: Irradiation regimen of 28 sessions in IMRT to the pelvis concurrent with CT, followed by 4 BATD applications. VMAT QTRT28 + BATD: Irradiation regimen of 28 sessions in VMAT to the pelvis concurrent with CT, followed by 4 BATD applications. One American Dollar $ is equivalent to 3.8 Peruvian Sol (S/)
